# Multiple Mass Lesions in Pneumocystis Pneumonia

**DOI:** 10.7759/cureus.21590

**Published:** 2022-01-25

**Authors:** Misato Kobayashi, Yukari Tsubata, Yohei Shiratsuki, Takamasa Hotta, Takeshi Isobe

**Affiliations:** 1 Department of Internal Medicine, Division of Medical Oncology & Respiratory Medicine, Shimane University Faculty of medicine, Izumo, JPN; 2 Department of Internal Medicine, Division of Medical Oncology & Respiratory Medicine, Shimane University Faculty of Medicine, Izumo, JPN

**Keywords:** mycosis fungoides, multiple mass lesions, ground-glass opacity, grocott’s staining, immunocompromised host, pneumocystis pneumonia, pneumocystis jirovecii

## Abstract

We encountered a case of pneumocystis pneumonia (PCP) presenting with multiple mass lesions in a human immunodeficiency virus (HIV)-negative patient. Diagnosis of PCP before bronchoscopy was difficult because chest computed tomography (CT) findings were atypical of PCP and a serum (1,3)-β-D-glucan concentration was within normal limits. Bronchoscopic biopsy and Grocott’s staining enabled the diagnosis of PCP. PCP can show various patterns on chest CT images, depending on the immune status of the host. In high-risk patients, such as those who are immunocompromised, bronchoscopy should be performed with suspected cases of PCP, even if CT imaging does not show typical ground-glass opacity.

## Introduction

Although pneumocystis pneumonia (PCP) typically shows bilateral ground-glass opacity (GGO) on chest computed tomography (CT) images, it can present with various patterns depending on the patient’s immune status [1-3]. As atypical examples, nodules or mass lesions are sometimes reported in human immunodeficiency virus (HIV)-positive patients, but less commonly (7.2%) in HIV-negative patients [1]. In addition, the sensitivity of serum (1,3)-β-D-glucan (β-D-glucan) concentration for PCP is known to be as high as 94.8%-96% [4,5]. Here, we report a case of β-D-glucan-negative PCP presenting with multiple lung mass lesions without GGO.

## Case presentation

**Figure 1:**
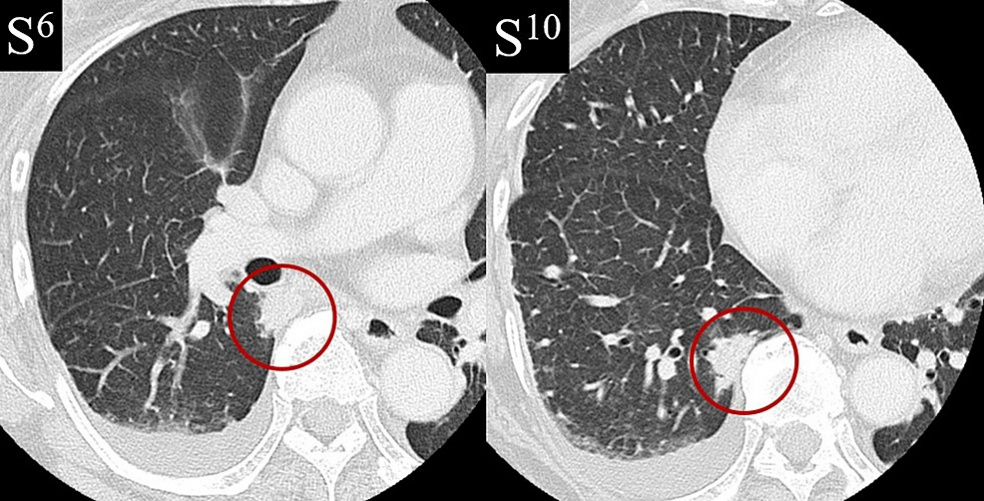
Contrast-enhanced CT revealed no source of fever other than two mass lesions (18×11 mm and 26×10 mm) in S6 and S10 in the right lower lobe. Pleural effusion, which may be influenced by inflammation, was also observed in the right lung.

**Figure 2:**
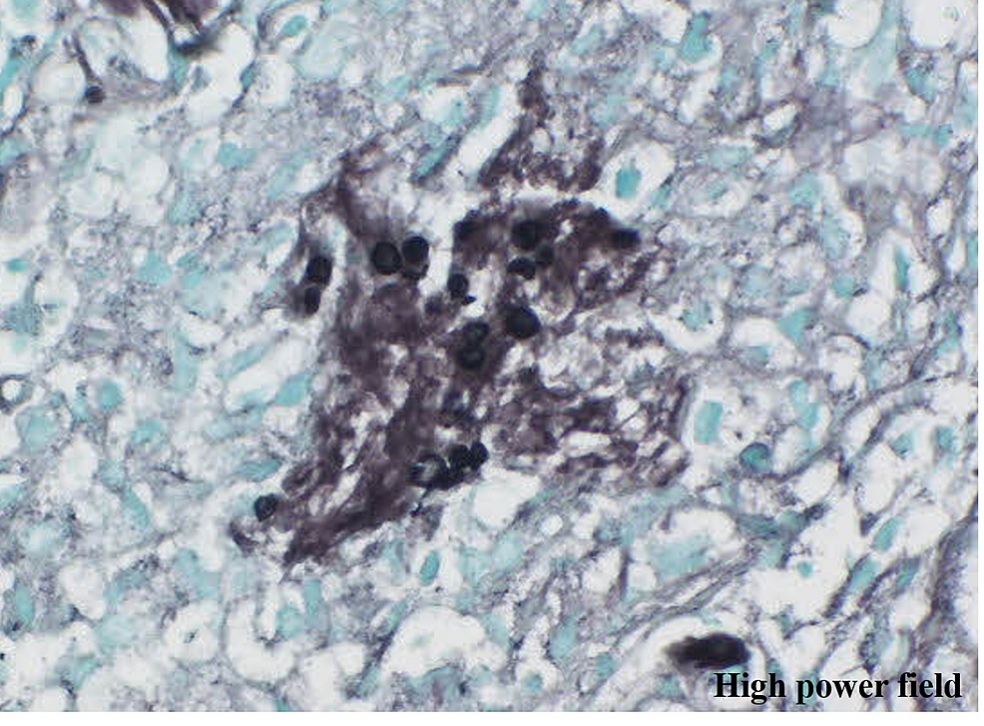
Grocott–Gomori's methenamine silver stain demonstrating cysts of classic Pneumocystis jirovecii.

A 69-year-old woman undergoing treatment for mycosis fungoides with biweekly pirarubicin hydrochloride, oncovin, cyclophosphamide, and prednisolone experienced fever on day 7 of the fifth chemotherapy course. Initial chest CT images were unremarkable, and serum procalcitonin and β-D-glucan levels were within normal limits. On suspicion of bacterial or fungal infection, we administered meropenem and amphotericin B, which proved ineffective. Approximately 10 days after the initial chest CT, we took contrast-enhanced CT. We found no source of fever other than two mass lesions in S6 and S10 in the right lower lobe (Figure 1). Pleural effusion, which may be influenced by inflammation, was observed in the right lung. The patient was asymptomatic, and her SpO_2_ level was within normal limits. Bronchoscopy was performed, and Grocott’s staining of bronchial biopsy specimens revealed cysts of Pneumocystis jirovecii (Figure 2).

 

**Figure 3:**
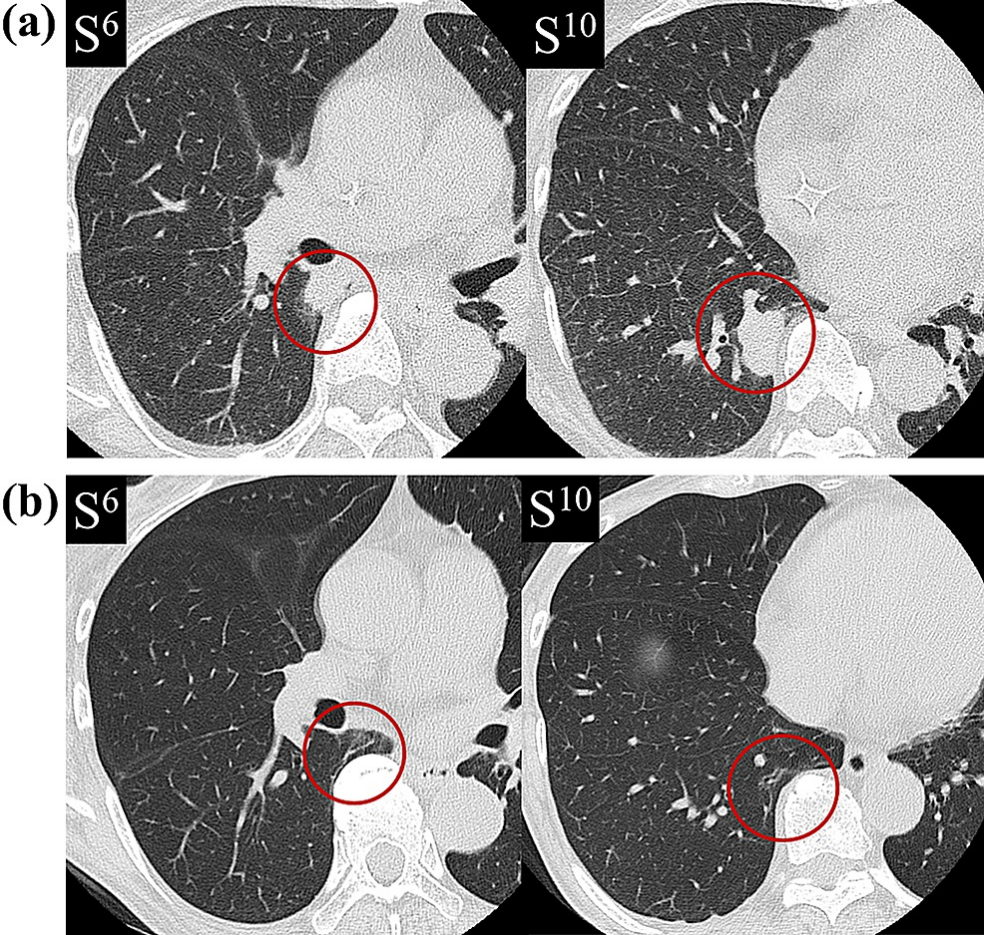
Chest CT before and after treatment. (a) Nine days after the previous CT examination, the masses became enlarged (20×13 mm and 28×19 mm). (b) CT showed the disappearance of the masses four months after starting treatment.

We diagnosed PCP 4 days after bronchoscopy, during which the chest CT showed enlargement of the masses (Figure 3a). A combined regimen of trimethoprim and sulfamethoxazole was initiated, but the medication was changed to atovaquone (1,500 mg/day) because of hyperkalemia. The treatment was continued for a total of three weeks; the fever resolved, and all mass lesions diminished (Figure 3b). Grocott’s staining was important for the diagnosis in this case.

## Discussion

We encountered a rare case of PCP in an HIV-negative patient with multiple lung mass lesions. A typical chest CT image of PCP shows bilateral GGO [1-3], and even if nodules or mass lesions are observed, they are often surrounded by GGO [6-8]. Nodules or mass lesions have been reported to reflect granulomas [6], and our patient was considered to have some degree of sustained immune function against infection. Pneumocystis jirovecii is notably difficult to detect in specimens from HIV-negative patients, who have a stronger immune response to Pneumocystis but fewer pathogens when compared to HIV-positive patients [9]. The polymerase chain reaction from bronchoalveolar lavage fluid is useful for the detection of Pneumocystis jirovecii [10-12]. However, in this case, biopsy and Grocott’s staining proved to be the most important steps for an accurate diagnosis because the lesions were localized.

PCP was not included in the differential diagnosis of this case because the chest CT findings were atypical, and the patient’s serum β-D-glucan level was within normal limits. Tasaka et al. reported that serum β-D-glucan level was the most reliable PCP indicator among the serum levels of lactate dehydrogenase, β-D-glucan , Krebs von den Lungen-6 (KL-6), and C-reactive protein in a retrospective analysis of patients with PCP diagnosed using bronchoalveolar lavage [13]. In the above-mentioned study, the cutoff value of the β-D-glucan concentration for PCP was 31.1 pg/mL with a sensitivity of 92.3% and specificity of 86.1%. A previously published meta-analysis showed a sensitivity of 94.8% and specificity of 86.3% for β-D-glucan concentration in patients with PCP [5]. However, the reference range for β-D-glucan differs depending on the assay method employed. Although the β-D-glucan concentration was within normal limits in this case, which may reflect the small number of pathogens, sufficient data have not been obtained to determine whether the β-D-glucan concentration reflects the burden of Pneumocystis in the lung.

Due to the diversity of background and clinical findings, diagnosis of PCP is more likely to be delayed in HIV-negative patients than in HIV-positive patients, resulting in delays in the therapeutic intervention [12]. As PCP presents various imaging features, such as bilateral GGO, consolidation, cysts, and nodules [1-3], depending on the patient’s immune status, it is important to consider PCP regardless of CT image patterns if a patient is at risk of opportunistic infections.

## Conclusions

PCP can present with various chest CT findings, depending on the patient’s immune status. PCP is more common in immunocompromised patients and can lead to life-threatening conditions. It is important to consider PCP even when chest CT findings are atypical, such as multiple nodules, and to attempt bronchoscopy to diagnose PCP if the patient’s condition allows. A prompt diagnosis and early anti-PCP treatment are necessary in suspected cases of PCP.
